# Real-world study of the impact of recurrent/metastatic squamous cell carcinoma of the head and neck (R/M SCCHN) on quality of life and productivity in Europe

**DOI:** 10.1186/s12885-021-08557-2

**Published:** 2021-07-24

**Authors:** Prianka Singh, Bryan Bennett, Tom Bailey, Gavin Taylor-Stokes, Ivana Rajkovic, Marta Contente, Sharon Curtis, Chris Curtis

**Affiliations:** 1 Bristol Myers Squibb, 3401 Princeton Pike, Lawrence, NJ 08648-1205 USA; 2 Bristol Myers Squibb, Uxbridge Business Park, Sanderson Road, Uxbridge, Middlesex, UB8 1DH UK; 3 Adelphi Real World, Grimshaw Lane, Bollington, Cheshire, SK10 5JB UK; 4 The Swallows Head & Neck Cancer Support Group, The Michael Stenhouse Centre, 68-70 Waterloo Road, South Shore, Blackpool, FY4 1AB UK

**Keywords:** Squamous cell carcinoma of the head and neck, SCCHN, First line treatment, Platinum-eligible, Quality of life, EQ-5D, FACT-H&N, WPAI, Europe

## Abstract

**Background:**

Although current therapy for patients with early-stage squamous cell carcinoma of the head and neck (SCCHN) is potentially curative, the recurrence rate is high. Patients with recurrent or metastatic (R/M) SCCHN have a poor prognosis and substantial disease burden, including impaired health-related quality of life (HRQoL), productivity loss and indirect costs, such as need for caregiver support. The aim of this study was to characterize the impact of R/M SCCHN and its first-line treatment on patient and caregiver quality of life, daily activities and work productivity using real-world evidence from Europe.

**Methods:**

This was a multicentre retrospective study of patients with R/M SCCHN in France, Germany, Italy, Spain and the United Kingdom incorporating patient and caregiver surveys, and a physician-reported medical chart review, conducted between January and May 2019. Patients aged 18 or over with a physician confirmed diagnosis R/M SCCHN completed four validated measures of disease activity and its impact on quality of life and work productivity, while caregivers also completed questionnaire to assess the burden of providing care. Physicians provided data for clinical characteristics, patient management, testing history and treatment patterns.

**Results:**

A total of 195 medical/clinical oncologists provided data for 937, predominantly male (72%) patients, with almost half of patients aged over 65 years. The most frequently reported symptoms were fatigue (43%), weight loss (40%), pain (35%) and difficulty swallowing (32%). The EXTREME regimen was the most common first line therapy in over half of patients, who reported moderate or extreme pain/discomfort, and anxiety/depression, and problems with self-care resulting in a diminished health status compared with the general population. Only 14% were employed with high absenteeism or presenteeism, and over half of patients had a caregiver for whom the burden of care was substantial.

**Conclusion:**

Our results provide real-world insight into the multi-faceted burden associated with R/M SCCHN. The combination of poor HRQoL and the impairment in daily activities, social life and employment illustrates the wider impact of R/M SCCHN on patients and their caregivers, and highlights a need for novel 1 L treatment regimens to improve the humanistic and productivity burdens of this cancer.

**Supplementary Information:**

The online version contains supplementary material available at 10.1186/s12885-021-08557-2.

## Background

Head and neck cancer is the sixth most common malignancy globally [1], and comprises tumours originating in a range of sites including the oral cavity, pharynx, larynx, nasal cavity, and salivary glands. The majority of tumours arise from squamous cells on the epithelial surfaces and are therefore referred to as squamous cell carcinomas of the head and neck (SCCHN) [2]. In Europe, head and neck cancer accounts for around 4% of all cancers, amounting to 139,000 new cases per year, of which more than 90% are SCCHN [3].

Due to the heterogeneity of anatomic sites affected and the significant symptom burden, treatment is complex and requires a multidisciplinary team of specialists [1, 4, 5] as patients may require reconstructive surgery, nutritional support, speech and language therapy, and psychological support in addition to systemic therapy [6]. Although patients with localized SCCHN can be treated with potentially curative therapy including surgery, radiation therapy, chemotherapy, and/or biologic therapy, the recurrence rate in early stage SCCHN is 10–20%, and in locally advanced SCCHN is approximately 50% [7].

Patients with recurrent or metastatic (R/M) SCCHN have a poor prognosis, with a median overall survival of less than one year [7]. The treatments for patients with R/M disease in the first line (1 L) setting are limited [8] and, although a number of platinum-based therapies exist, not all patients respond to, or are tolerant of, platinum. For those patients who are platinum-eligible, the 1 L treatment that is more frequently recommended for R/M SCCHN is the cetuximab-based, EXTREME regimen (cetuximab + platinum + fluorouracil) [9].

In addition to a poor prognosis, patients with R/M SCCHN experience a significant impact on health-related quality of life (HRQoL) and productivity from both the disease and its treatment, with physical implications causing greater psychological distress than other cancer types [10, 11]. Moreover, factors such as oral dysfunction, loss of appetite, reduced social functioning and high levels of anxiety are barriers to patients with SCCHN to return to work and normal social activity after treatment [12], and work impairment has been associated with reduced wellbeing and HRQoL in other cancers [13, 14].

Not only is head and neck cancer associated with reduced odds of being in employment and decreased earnings for those who are employed [15, 16], but the productivity losses associated with time off work or reduced work hours due to head and neck cancer are substantial [16]. The indirect costs due to reduced work productivity, such as loss of income and need for caregiver support, are also relevant for understanding the societal burden of head and neck cancers.

To date, few studies have investigated work productivity and indirect costs for head and neck cancer, and a 2014 systematic literature review on the economic burden of head and neck cancer identified only four studies reporting indirect costs [1]. Accordingly, the impact of cancer on work productivity has often been overlooked in the value frameworks produced by major organizations such as the American Society of Clinical Oncology (ASCO) and the European Society of Medical Oncology [3, 17, 18]. The value frameworks that have recently been proposed in response to high prices of cancer treatment are intended to assist payers and health systems in their coverage and payment decisions. These use multiple criteria such as clinical efficacy, toxicity, cost, innovation, burden of illness, patient and societal burden, and ethical and equity considerations to assess the value of oncology treatments, although no single model encompasses the components needed for a full societal assessment [17].

The aim of this study was to generate real-world evidence describing the impact of R/M SCCHN and its treatment on patient and caregiver quality of life, daily activities and work productivity in Europe, and to highlight the humanistic and economic burden of R/M SCCHN on patients, their caregivers, and society as a whole. This study focused specifically on patients with R/M SCCHN who were eligible to receive platinum-based chemotherapy in a 1 L setting, in order to strengthen the understanding of the multi-faceted burden within this specific patient group.

## Methods

### Study design

This was a multicentre retrospective study of patients with R/M SCCHN in five European counties (France, Germany, Italy, Spain and the United Kingdom [UK]) conducted between January and May 2019, incorporating patient and caregiver surveys, and physician-reported medical chart review data.

To be invited to take part in the study, specialists in medical/clinical oncology or otolaryngology (Germany only) must have been in clinical practice for more than five and less than 35 years, have a caseload at the point of enrolment of at least ten R/M SCCHN patients, and involved in treatment decisions for patients with R/M SCCHN. Participating physicians included in the study were each invited to recruit up to ten consecutive patients diagnosed with R/M SCCHN.

To be eligible, patients had to be aged 18 or over with a physician confirmed diagnosis of R/M SCCHN with either Stage III (locally advanced with recurrence) or Stage IV (metastatic) disease, with primary tumour site in the oral cavity (lip, tongue, gum, floor of mouth and other parts of mouth), oro/hypopharynx or larynx. Patients included in the study were either platinum-naïve, newly diagnosed with metastatic disease, or were previously treated and platinum sensitive, with progression or recurrence on or beyond six months of last platinum therapy dose.

### Data collection

Eligible patients were asked to complete four validated patient reported outcome (PRO) measures of disease activity and its impact on HRQoL and productivity at the point of enrolment. These included the European Quality of Life Five Dimension Questionnaire (EQ-5D-3L) [19, 20], the Functional Assessment of Cancer Therapy–General (FACT-G) [21], the Functional Assessment of Cancer Therapy–Head and Neck Cancer (FACT-H&N) [22] and the Work Productivity and Activity Impairment questionnaire [23].

The EQ-5D-3L questionnaire is a self-reported measure of generic health that consists of five dimensions (mobility, self-care, usual activities, pain/discomfort, and anxiety/depression), each with three levels of health severity that can describe 243 unique health states. A number of societal value sets have been derived from population-based valuation studies that, when applied to the health state vector, result in a preference- based score that ranges from states worse than dead (< 0) to 1 (full health), anchoring dead at 0. In addition, the measure includes a visual analogue scale (VAS) on which health is rated on a scale from 0 (worse imaginable health) to 100 (best imaginable health). Scores on the EQ-5D VAS were compared with reference values from the individual population norms [24] and the minimally important difference (MID) for a change of 7in the VAS score and 0.08 in the utility index [25].

The FACT-G is a 27-item instrument containing four subscales: Physical Well-Being (PWB; 7 items); Functional Well-Being (FWB; 7 items); Social Well-Being (SWB; 7 items); and Emotional Well-Being (EWB; 6 items). Each subscale is a 5-point Likert-type scale ranging from 0 (not at all) to 4 (very much) with a recall period of the past 7 days [26]. The FACT-H&N consists of an additional 9-items appended to the FACT-G to create a head and neck cancer specific subscale [27]. Scores are calculated separately for each domain, and an unweighted summary score is calculated for the FACT-G and the total FACT-H&N with higher scores reflecting better HRQoL. The highest possible score is 28 for the PWB, SWB, and FWB subscales, 24 for the EWB subscale, 108 for the FACT-G total score, and 144 for the total FACT H&N.

The WPAI is a validated, non-disease-specific tool and consists of six items used to derive four domains including work time missed (absenteeism), impaired productivity at work (presenteeism), overall work impairment (combined absenteeism and presenteeism), and impairment in non-work-related activities due to health problems (activity impairment), over the previous seven days. The WPAI outcomes are expressed as impairment percentages, with higher numbers indicating greater impairment and less productivity [28].

For patients who had a caregiver (formal or informal), the caregivers were also invited to complete a questionnaire to assess the burden of providing care as measured by the Zarit Burden Interview (ZBI). The ZBI is a 22-item questionnaire that is used to assess the level of burden experienced by the caregiver. The ZBI is scored from 0 to 88 with a higher score indicating a higher or more severe burden [29]. Details of the caregivers’ productivity (WPAI) and health status (EQ-5D-3L) were also recorded in the caregiver survey.

In addition to completing validated PRO measures, patients and caregivers were asked to score a number of impact statements, for example patient-reported impact on daily activities and caregiver-reported impact of caregiving on health, on a scale of 1 to 7, where 1 indicates ‘No impact’ and 7 indicates ‘Extremely high impact’. Upon return of the patient questionnaire, physicians were required to record data for each corresponding patient on an electronic case report form (eCRF) that included demographics, clinical characteristics and patient management, symptoms, comorbidities, treatment history and side effects.

### Data analysis

Reference values from studies in patients with different forms of advanced cancer were used to compare scores for the FACT-H&N, FACT-G and its subscales [30, 31]. HRQoL, as measured by the FACT-H&N, was grouped into quartiles, with the lowest quartile representing patients with the poorest HRQoL and the highest quartile representing patients with the highest HRQoL [32, 33]

Categorical variables were described by counts and proportions (average) of respondents, and continuous numerical variables were described by means and standard deviations. Missing data in the patient questionnaire were not explicitly addressed (e.g. imputed) but the base (n) for each variable was reported to enable calculation of the number of patients missing data.

## Results

A total of 195 medical/clinical oncologists (including otolaryngologists in Germany – see Supplementary Table 1) provided data for 937 patients, 577 of whom (France 114, Germany 117, Italy 130, Spain 117 and the UK 99) were eligible to receive platinum-based treatment first line (86% were platinum naïve and 14% platinum sensitive). Prior to the diagnosis of recurrent/metastatic SCCHN, almost half of the patients (47%) had received radiotherapy, one third (33%) had received surgery, 17% pharmacological therapy, 1% best supportive care only, and one third (33%) were untreated. The patient population was predominantly male (72%) with similar distribution of patients aged under 65 (54%) and over 65 (46%) years and a mean [SD] age 62.8 [8.5] years (Table 1).

**Table 1:** Demographic details of 1 L platinum eligible patients with R/M SCCHN

	Overall	France	Germany	Italy	Spain	UK
Number of physicians	195	31	66	50	32	16
Number of patients	577	114	117	130	117	99
Number of caregivers	238	81	53	21	38	45
Age						
Mean (SD) years	62.8 (8.5)	62.9 (8.6)	64.2 (7.2)	61.6 (9.6)	60.8 (8.6)	64.9 (7.4)
Under 65 years (%)	53.6	50.0	50.4	60.8	63.2	40.4
65 years and over (%)	46.4	50.0	49.6	39.2	36.8	59.6
Gender (%)						
Male	72.3	83.3	64.1	72.3	72.6	68.7
Staging at initial diagnosis (%)						
Stage I	1.0	1.8	1.7	0.8	0.9	0.0
Stage II	10.9	6.1	6.0	17.7	14.5	9.1
Stage III	23.2	27.2	2.6	36.9	30.8	16.2
Stage IVA	11.4	12.3	6.8	11.5	19.7	6.1
Stage IVB	5.9	3.5	9.4	8.5	4.3	3.0
Stage IVC	47.3	49.1	73.5	24.6	29.9	64.6
Current staging (%)						
Stage III	5.7	2.6	3.4	12.3	7.7	1.0
Stage IVA	14.7	21.9	0.9	20.0	20.5	9.1
Stage IVB	13.9	12.3	8.5	20.8	17.9	8.1
Stage IVC	65.7	63.2	87.2	46.9	53.8	81.8
ECOG score at initial diagnosis (%)						
0–1	86.2	76.3	95.8	80.0	84.6	96.0
0	27.4	11.4	47.9	28.5	22.2	26.3
1	58.8	64.9	47.9	51.5	62.4	69.7
2+	13.5	23.7	4.3	18.5	15.4	4.0
Current ECOG score (%)						
0–1	78.6	59.6	88.0	76.1	80.3	89.9
0	12.7	2.6	18.8	12.3	8.5	22.2
1	65.9	57.0	69.2	63.8	71.8	67.7
2	21.5	40.4	12.0	23.8	19.7	10.1
Primary tumour site (%)						
Oral cavity	31.9	28.1	35.9	31.5	34.2	29.3
Oropharynx	26.7	38.6	17.9	20.0	23.1	36.4
Hypopharynx	17.0	21.1	12.0	13.1	18.8	21.2
Larynx	23.9	12.3	33.3	34.6	23.9	12.1

ECOG = Eastern Cooperative Oncology Group performance status

The main disease symptoms reported by physicians included fatigue (43%), weight loss (40%), pain (35%), difficulty swallowing (32%), anorexia (23%) and dry or persistent sore throat (19%). The overall Charlson Comorbidity Index was 5.2, with hypertension (36%), chronic pulmonary disease (18%), anxiety (14%), depression (10%) and mild liver disease (10%) being the most common comorbidities.

The majority (79%) of patients had an Eastern Cooperative Oncology Group (ECOG) performance status score between 0 (fully active) and 1 (restricted in physically strenuous activities) at enrolment, and two thirds (66%) were classified as having metastatic disease (stage IVC). The oral cavity was the most common primary tumour site (32%) overall, although the oropharynx was the more frequent site in France (39%) and the UK (36%). The mean time from SCCHN diagnosis to diagnosis of metastatic disease was 4.4 months, with the lung and lymph nodes being the main sites of metastatic disease (in 70 and 55% of patients, respectively).

### Treatment regimen

The EXTREME regimen was the most common therapy prescribed for over half (52%) of all patients at 1 L, whilst 9% received a separate cetuximab-based regimen (Table 2). Platinum based (mono- and combination, excluding EXTREME) therapy was prescribed to over one third (35%) of patients, with immunotherapy used in only 3% of patients, most commonly used in Germany (6% of patients).

**Table 2:** Distribution of first line treatment in platinum-eligible patients with R/M SCCHN (%)

	Overall (***n*** = 577)	France (***n*** = 114)	Germany (***n*** = 117)	Italy (***n*** = 130)	Spain (*n* = 117)	UK(***n*** = 99)
EXTREME	52	64	45	38	55	61
Cetuximab based	9	10	7	15	11	3
Platinum monotherapy	3	1	8	3	4	0
Platinum plus taxane (+/− other)	19	19	20	26	17	9
Platinum plus other (excluding taxane)	13	2	14	16	10	24
Immunotherapy	3	4	6	1	2	3
Other	1	0	1	1	1	0

Overall, 95 patients (16%) went on to receive a second line of therapy, with almost two thirds (62%) of these patients receiving PD-1 immunotherapy. Almost one half (42%) of patients experienced side effects from their current 1 L SCCHN drug treatment, most commonly appetite loss (20%), asthenia (18%), fatigue (17%), anaemia (15%) and nausea (14%).

### Patient reported outcomes

**Fig. 1:**
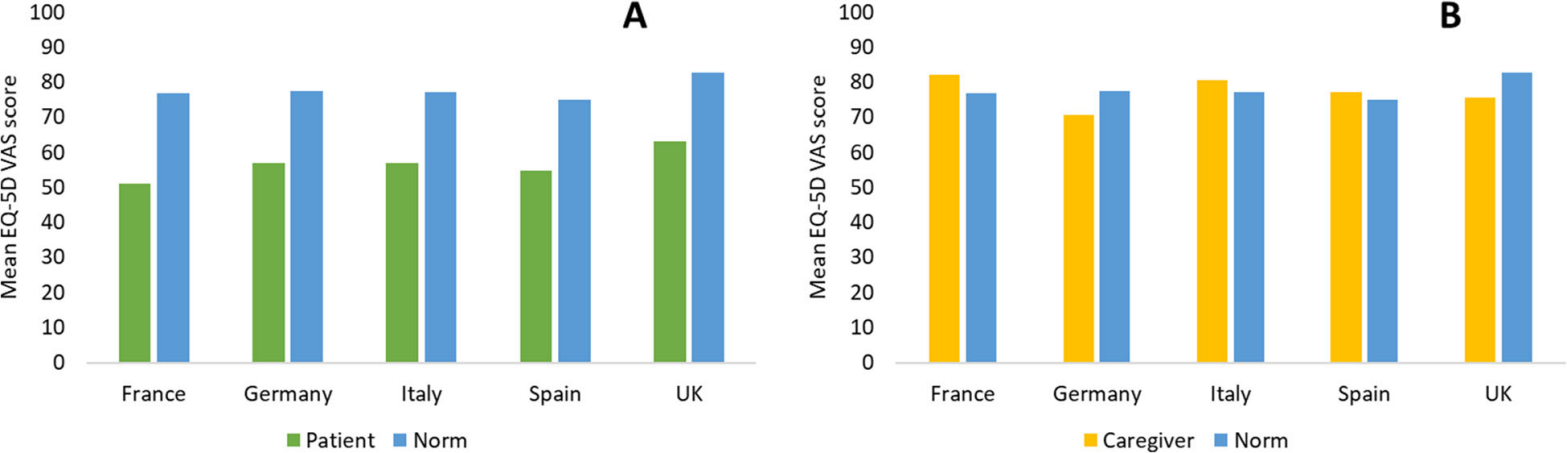
Mean EQ-5D VAS scores for platinum-eligible patients with R/M SCCHN (**A**) and their caregivers (**B**) showing national population norms

Patients reported a diminished health status, as indicated by the EQ-5D-3L, with a mean utility score of 0.570 and a mean VAS of 56.4 (Fig. 1A) which was significantly lower than the reference norm values of the national populations [24]. Patients also reported moderate or extreme pain/discomfort (78%), moderate or extreme anxiety/depression (71%) and most patients reported at least some problems with self-care (41%) and performing usual activities (62%).

**Fig. 2:**
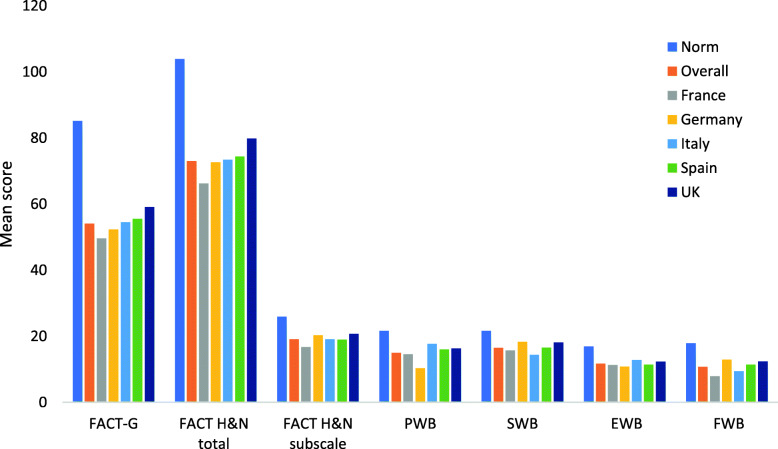
Patient scores on FACT-G and FACT H&N total and subscale scores overall, by country and reference value^†^

The overall mean score for the FACT-G (54.1) was substantially lower than the reference normative value reported for all cancers (80.9) [30] with the lowest mean score (49.6) in France and highest (59.1) in the UK (Supplementary Table 2; Fig. 2). The mean FACT-G score for patients receiving EXTREME or cetuximab-based regimens was slightly lower than the overall population score at 53.3. Patients receiving platinum-based combination (excluding taxane) and EXTREME regimens reported greatest impact of side effects according to the FACIT-GP5 (“I am bothered by side effects of treatment”), with 69 and 65%, respectively, indicating “Somewhat”, “Quite a bit” or “Very much” impact.

The mean score for the total FACT-H&N scale of 73.2 was also substantially lower than the reference normative value (103.9) (31), with mean scores ranging from 66.2 in France to 79.8 in the UK. Similarly, the mean score of 19.1 recorded on the FACT-H&N subscale was low compared with a reference normative value of 25.9, with the lowest mean score of 16.7 recorded in France (Supplementary Table 3; Fig. 2). All subscale scores were substantially lower than the reference norms for a general head and neck cancer population [31], with similar distribution across all countries (Fig. 2).

### Employment status

At diagnosis of SCCHN, only 5% of patients were on sick leave compared with over a quarter (28%) of patients on enrolment into the study with R/M SCCHN (Table 3). Consequently, over one-third (37%) of patients were in full- or part-time employment at diagnosis compared with only 14% on enrolment with R/M disease (Table 3). The age of retirement (i.e. receipt of statutory pension) varies from 65 years (Germany, Spain and the UK) to 66 years in France and 67 years in Italy. Over a third (40%) of working patients reported an average reduction of 39 working hours per month due to R/M SCCHN, while ‘allowing a phased return’ (67%) was the most frequently reported expected adjustment to be made when returning to work. The number of retired patients with R/M SCCHN was comparable at diagnosis (44%) with the status at enrolment (46%).

**Table 3:** Employment status (%) of 1 L platinum eligible patients at diagnosis and at enrolment into the study with R/M SCCHN

	Diagnosis (***n*** = 571)						Current (***n*** = 548)					
	Overall	France	Germany	Italy	Spain	UK	Overall	France	Germany	Italy	Spain	UK
Working full time	30	33	28	38	35	13	8	8	10	12	7	1
Working part time	7	2	7	10	6	8	6	2	4	7	10	5
Long term sick leave	5	4	10	5	6	0	28	31	29	29	31	15
Homemaker	5	2	3	8	7	4	4	1	3	6	6	3
Retired	44	52	44	32	34	61	46	52	44	40	35	63
Unemployed	9	8	9	6	12	13	8	6	9	6	9	12

### Work productivity and activity impairment

**Fig. 3:**
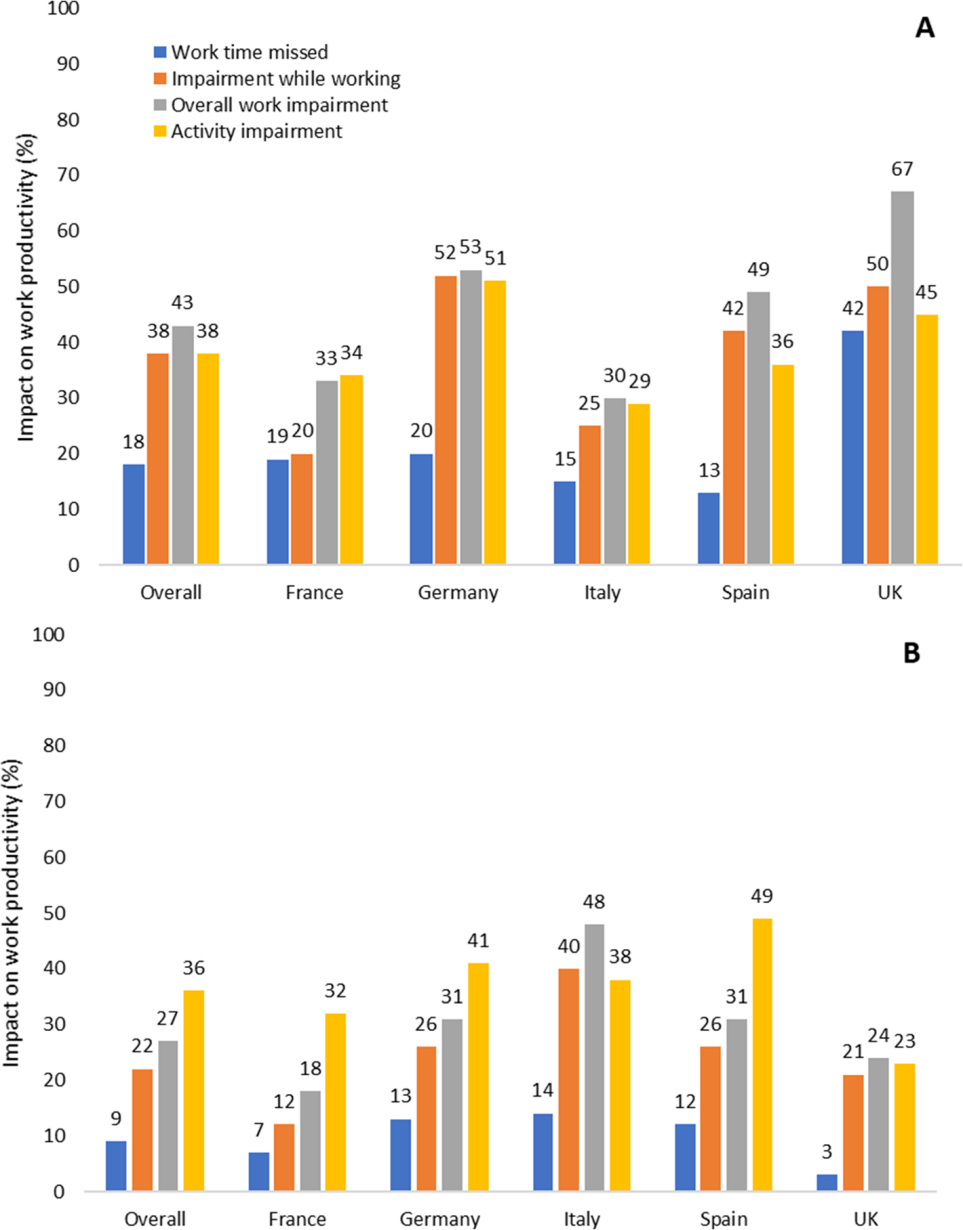
Patients (**A**) and caregiver (**B**) work productivity and non-work activity impairment

Overall, the impact of R/M SCCHN on patients’ ability to work was considerable, with those patients still in employment attributing an overall work impairment, according to the WPAI, of 43% to their disease. On average, patients in employment reported that almost one fifth (18%) of work time was missed due to R/M SCCHN (absenteeism), together with considerable impairment at work and an average presenteeism of 38%. The overall activity impairment (excluding work) attributed to R/M SCCHN for all patients was considerable, with patients reporting a mean score of 38% (Fig. 3A).

### Impact on activities of daily living and employment

**Fig. 4:**
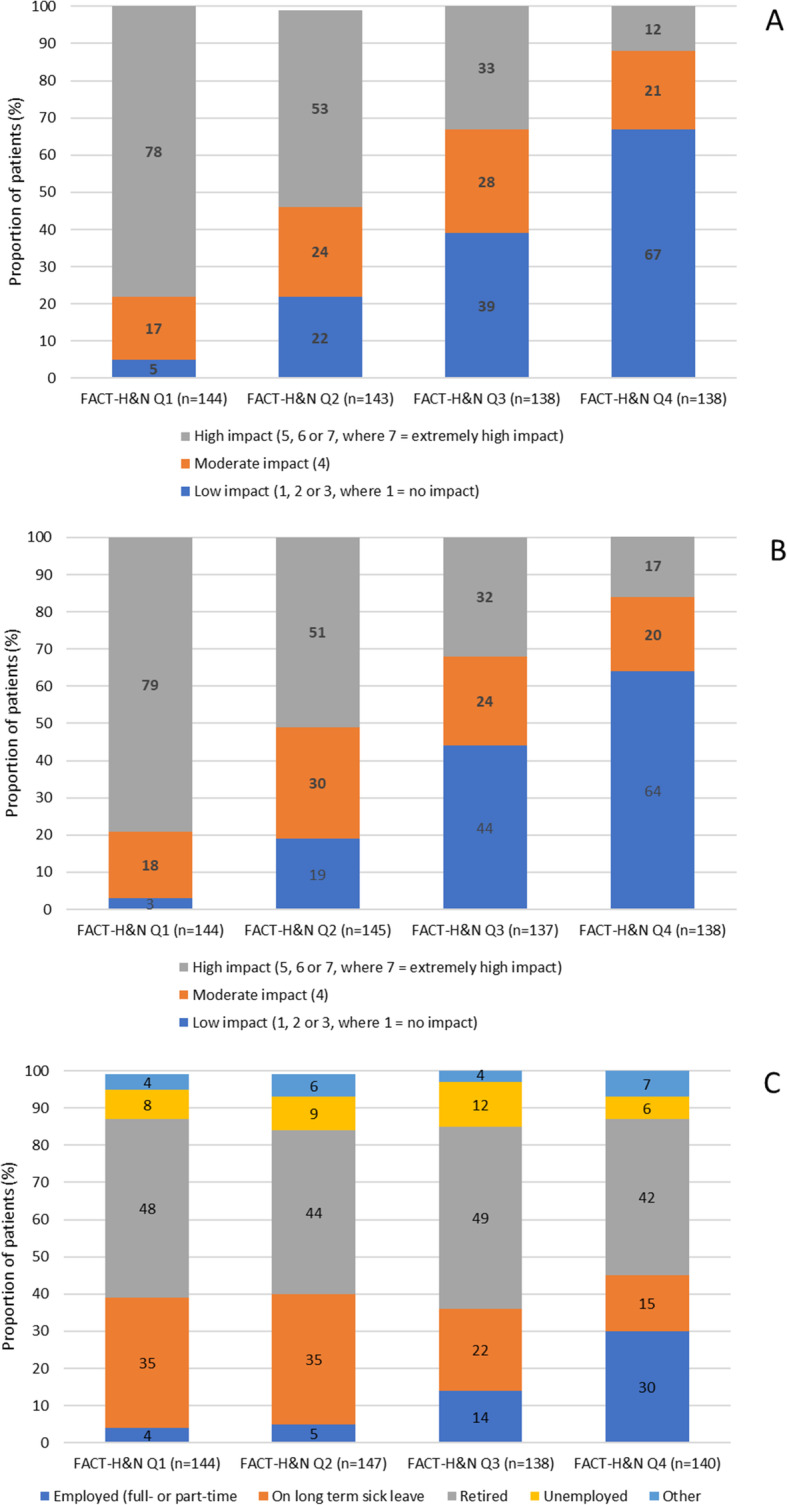
Impairment of disease on activities of daily living (**A**), social activities (**B**) and employment status (**C**)

Just under half (44%) of 1 L platinum eligible patients with R/M SCCHN reported a high level of interference in their ability to perform activities of daily living (as indicated by a score of 5–7 where 7 is ‘extremely high impact’), with a similar proportion (45%) of patients reporting high interference in their social activities. Patients with the poorest HRQoL (lower quartile) reported the highest level of interference in their daily activities (78%), compared with 12% patients with the highest HRQoL (upper quartile) [Fig. 4A], and a similar trend in relation to social activities (79% vs 17%) [Fig. 4B].

There was a clear relationship between patients’ HRQoL (as indicated by the FACT-H&N) and patients’ working status post diagnosis of R/M SCCHN: only 4% of patients with the poorest HRQoL (lowest quartile) were in employment compared with 30% with the highest HRQoL (highest quartile) who were working either full-or part-time (Fig. 4C). Patients with the poorest HRQoL (lowest quartile) were more likely to be on long-term sick leave (35%) than patients with the highest HRQoL (highest quartile), in which less than a fifth (15%) of patients were on sick leave (Fig. 4C).

### Caregiver burden

**Fig. 5:**
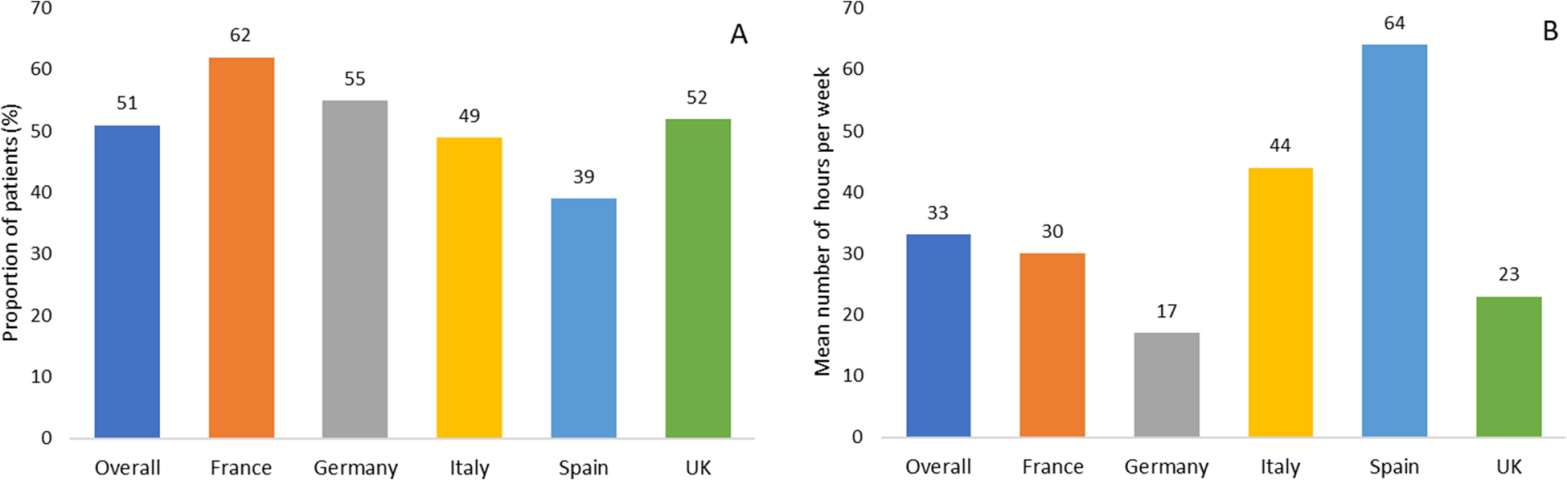
Proportion of patients with R/M SCCHN who require a caregiver (**A**) and time spent (hours/week) by caregivers supporting their patients (**B**)

On average, over half (51%) of patients had a caregiver to support their daily needs (Fig. 5A) with the highest reported in France (62%) and least in Spain (39%). Caregiver support was greater in patients aged 65 and above (55%) in comparison with those aged under 65 (48%). On average, one third (35%) of caregivers reported spending over 30 h per week providing emotional and physical support to patients. This was particularly high in Italy and Spain (44 and 64 h, respectively; Fig. 5B).

Consequently, over one quarter (27%) of caregivers reported an impairment in their work productivity as a result of the care they provided, and over one third (36%) also reporting impairment in their daily activities, with highest impairment (49%) in Spain (Fig. 3B).

Although caregivers were generally in good health, as confirmed by the overall EQ-5D VAS (77.4) score (Fig. 1B), over half (51%) reported that they were moderately/extremely anxious or depressed. Nearly one half (49%) of caregivers also reported that providing emotional support and encouragement to the patient was the most troublesome activity of caregiving. With a mean ZBI score of 36, the greatest proportion of caregivers reported a ‘mild to moderate burden’ in caring for patients, while almost two-fifths (39%) of caregivers reporting a ‘moderate/severe burden’, with the ZBI scores indicating that 81% were at risk of depression [34], and ‘personal strain’ contributing the greatest level of burden. An association was observed between patient-reported HRQoL (via the FACT-H&N) and caregiver burden (via the ZBI); caregivers supporting patients with the poorest HRQoL (lower quartile) reported ‘moderate to severe’ caregiving burden (mean ZBI score of 42), in comparison with caregivers for patients with the highest HRQoL (upper quartile), who reported ‘mild to moderate’ caregiving burden (mean ZBI score of 27).

## Discussion

There are few data in the published literature comparing treatment strategies and outcomes in patients with R/M SCCHN across different countries [8]. This real world study showed similar approaches to the first-line management of R/M SCCHN across Europe, with similar outcomes. The burden of R/M SCCHN was highlighted by the poor health state and HRQoL reported by platinum-eligible patients receiving 1 L treatment, with a considerable reduction in the proportion of patients employed since diagnosis. Beyond the substantial patient impact, this study also demonstrated the clear burden of caregiving associated with R/M SCCHN, both with respect to the amount of time required to support patients and in terms of the emotional impact of providing care to this patient group.

The overall score on the FACT-G (54.1), which showed a significant impairment in HRQoL, was similar to reference values for other advanced cancers in Europe: patients with gastric cancer reporting the poorest FACT-G total score (49.4), followed by non small cell lung cancer (53.1), breast (53.7), melanoma (54.7), and prostate cancer (56.5) [35]. In addition to the considerable quality of life impact, this study highlighted the clear relationship between reduced HRQoL and restricted ability work, perform daily activities and socialize, highlighting the need to consider novel treatment approaches to improve HRQoL in R/M SCCHN. Even patients who were in employment at enrolment reported a high level of work impairment, indicating the debilitation associated with the disease. Over half of patients with R/M SCCHN required a caregiver to support their daily needs. Caregivers providing emotional and physical support to patients with R/M SCCHN experienced substantial impact on their own productivity and daily activities. Data from this study therefore highlights the multi-faceted, daily burden of R/M SCCHN experienced by 1 L platinum-eligible patients and their caregivers.

We acknowledge that there are some limitations in this non-interventional study, with physicians providing information on consecutive patients with R/M SCCHN. As patients and caregivers were encouraged, but not required, to complete all questions, the base sizes of responses fluctuate across different variables. Selection bias was also possible owing to the fact that the participating physicians surveyed represent a convenience sample that may not be representative of the overall population of physicians treating patients with R/M SCCHN. Moreover, eligible patients were selected by physicians on a consecutive basis from the point of physician enrolment into the study. It is therefore likely that patients who visited their physician more frequently were also more likely to have been included in the study.

Further to this, patient and caregiver participation was voluntary and therefore respondents unwilling or unable to participate are not represented in the sample, which may potentially result in the underrepresentation of patients with a particularly poor HRQoL (and their caregivers). The study, nevertheless, involved patients and caregivers recruited by a relatively high number of oncologists from different geographical regions thereby ensuring that the sample is likely to be representative of the overall population of patients with R/M SCCHN in those countries*.*

It is also important to note that reference values were used to indicate how the HRQoL data collected in this study compare with similar patient populations. Although efforts were made to identify a similar study which utilised the same PRO measure (FACT-H&N) for HRQoL, there were differences with regard to the study design and patient population, meaning interpretation should made with caution when comparing with the reference values. However, it is relevant and necessary to include reference values for these data to contextualise the results and highlight the particularly poor HRQoL seen in this 1 L R/M SCCHN patient population.

Finally, with regard to the wider interpretation of the impact on work and productivity reported in this study, we acknowledge that important country and regional differences exist in relation to societal policies and benefits, which may have implications on some of the responses recorded (such as the ability to take sick leave or availability of caregiver support). Further investigation is necessary to establish whether a correlation exists between health and societal policies and the subsequent impact on work and productivity in R/M SCCHN patients.

## Conclusions

This cross-sectional survey conducted in Europe characterizes the burden of R/M SCCHN for patients and their caregivers as substantial and multi-faceted with respect to HRQoL, health state, productivity and daily activity impairment. The combination of poor HRQoL and the consequential impairment in daily activities, social life and employment illustrates the wider impact on patients with particularly severe disease. The impact on caregivers in this population is also significant, with many at an increased risk of depression as a result of their caregiving role, while a clear relationship between patient-reported HRQoL and caregiving burden further demonstrates the impact of severe disease in this patient group. There is an unmet need for novel 1 L treatment regimens to improve clinical outcome for patients with R/M SCCHN and potentially address the substantial humanistic and productivity burdens of these patients and their caregivers.

## Supplementary Information

**Additional file 1: Supplementary Table 1.** Physician demographics. **Supplementary Table 2.** Patient scores on FACT-G and subscales overall, by country and reference normative value (30). **Supplementary Table 3.** Patient scores on FACT H&N questionnaire and subscales overall, by country and reference normative value (31).

## Data Availability

The datasets used and/or analysed during the current study are available from the corresponding author on reasonable request.

## Abbreviations

1 L: First line
ASCO: American Society of Clinical Oncology
ECOG: Eastern Cooperative Oncology Group
EQ-5D-3L: EuroQol 5 Dimension 3 Level Quality of Life Questionnaire
EWB: Emotional wellbeing
FACT H&N: Functional Assessment of Cancer Therapy – Head and Neck
FACT-G: Functional Assessment of Cancer Therapy – General
FWB: Functional wellbeing
HRQOL: Health related quality of life
MID: Minimally important difference
PRO: Patient reported outcome
PWB: Physical wellbeing
R/M: Recurrent/metastatic
SCCHN: Squamous cell carcinoma of the head and neck
SWB: Social/family wellbeing
UK: United Kingdom
VAS: Visual analogue scale
WPAI: Work Productivity and Activity Impairment
ZBI: Zarit Burden Interview
