# Supplementary material for: Real-world study of the impact of recurrent/metastatic squamous cell carcinoma of the head and neck (R/M SCCHN) on quality of life and productivity in Europe
Source: BMC Cancer. 2021 Jul 24;21:854. doi: 10.1186/s12885-021-08557-2 (PMC8310582; doi:10.1186/s12885-021-08557-2)
Supplement: Supplementary file 1 — Additional file 1: Supplementary Table 1. Physician demographics. Supplementary Table 2. Patient scores on FACT-G and subscales overall, by country and reference normative value (30). Supplementary Table 3. Patient scores on FACT H&N questionnaire and subscales overall, by country and reference normative value (31). [file 12885_2021_8557_MOESM1_ESM.docx]

**Supplementary Table 1. Physician demographics**

|  | Overall | France | Germany | Italy | Spain | UK |
| --- | --- | --- | --- | --- | --- | --- |
| N | 195 | 31 | 66 | 50 | 32 | 16 |
| Specialty (%) | | | | | | |
| Medical/clinical oncologist | 92 | 100 | 77 | 100 | 100 | 100 |
| Otolaryngologist | 8 | - | 23 | - | - | - |
| Year of qualification (%) | | | | | | |
| 1982-1994 | 17 | 13 | 21 | 14 | 19 | 19 |
| 1995-2004 | 44 | 55 | 56 | 26 | 44 | 31 |
| 2005-2013 | 38 | 32 | 23 | 60 | 38 | 50 |
| SCCHN caseload at point of enrolment, mean | 83 | 63 | 124 | 28 | 78 | 131 |
| R/M SCCHN caseload at point of enrolment, mean | 34 | 33 | 37 | 18 | 41 | 54 |
| Proportion of SCCHN practice time spent in a specialist cancer centre or other university / teaching hospital, (%) | 60 | 45 | 38 | 64 | 94 | 97 |

**Supplementary Table 2. Patient scores on FACT-G and subscales overall, by country and reference normative value (30)**

|  | Reference | Overall | France | Germany | Italy | Spain | UK |
| --- | --- | --- | --- | --- | --- | --- | --- |
| FACT-G | 80.9 | 54.1 | 49.6 | 52.3 | 54.5 | 55.5 | 59.1 |
| PWB | 21.3 | 15.0 | 14.6 | 10.3 | 17.7 | 16.0 | 16.3 |
| SWB | 22.1 | 16.5 | 15.7 | 18.3 | 14.4 | 16.6 | 18.1 |
| EWB | 18.7 | 11.7 | 11.3 | 10.8 | 12.8 | 11.4 | 12.3 |
| FWB | 18.9 | 10.7 | 7.9 | 12.9 | 9.4 | 11.4 | 12.4 |

**Supplementary Table 3. Patient scores on FACT H&N questionnaire and subscales overall, by country and reference normative value (31)**

|  | Reference | Overall | France | Germany | Italy | Spain | UK |
| --- | --- | --- | --- | --- | --- | --- | --- |
| FACT H&N total | 103.9 | 73.2 | 66.2 | 72.6 | 73.5 | 74.5 | 79.8 |
| FACT H&N subscale | 25.9 | 19.1 | 16.7 | 20.3 | 19.1 | 19.0 | 20.7 |
